# A Paper-Based Biomimetic Sensing Device for the Discrimination of Original and Fraudulent Cigarette Brands Using Mixtures of MoS_2_ Quantum Dots and Organic Dyes

**DOI:** 10.3390/bios13070705

**Published:** 2023-07-04

**Authors:** Fereshte Mohamadi Gharaghani, Sara Mostafapour, Bahram Hemmateenejad

**Affiliations:** 1 Chemistry Department, Shiraz University, Shiraz 71456-85464, Iran; fereshtemohamadi.chem@gmail.com (F.M.G.); mostafapoursara69@gmail.com (S.M.); 2 Medicinal and Natural Products Chemistry Research Centre, Shiraz University of Medical Sciences, Shiraz 71348-53734, Iran

**Keywords:** chemometrics, discrimination, electronic nose, MoS_2_ QDs, cigarette, volatile organic compounds

## Abstract

In this study, we investigated the combined effects of MoS_2_ QDs’ catalytic properties and the colorimetric responses of organic reagents to create a sniffing device based on the sensor array concept of the mammalian olfactory system. The aim was to differentiate the volatile organic compounds (VOCs) present in cigarette smoke. The designed optical nose device was utilized for the classification of various cigarette VOCs. Unsupervised Principal Component Analysis (PCA) and supervised Linear Discriminant Analysis (LDA) methods were employed for data analysis. The LDA analysis showed promising results, with 100% accuracy in both training and cross-validation. To validate the sensor’s performance, we assessed its ability to discriminate between five cigarette brands, achieving 100% accuracy in the training set and 82% in the cross-validation set. Additionally, we focused on studying four popular Iranian cigarette brands (Bahman Kootah, Omega, Montana Gold, and Williams), including fraudulent samples. Impressively, the developed sensor array achieved a perfect 100% accuracy in distinguishing these brands and detecting fraud. We further analyzed a total of 126 cigarette samples, including both original and fraudulent ones, using LDA with a matrix size of (126 × 27). The resulting LDA model demonstrated an accuracy of 98%. Our proposed analytical procedure is characterized by its efficiency, affordability, user-friendliness, and reliability. The selectivity exhibited by the developed sensor array positions it as a valuable tool for differentiating between original and counterfeit cigarettes, thus aiding in border control efforts worldwide.

## 1. Introduction 

Traditional sensing platforms work based on specific or highly selective chemical transducers. However, most of them show relative tendencies to a group of structurally similar compounds, an issue that is undesirable when measuring complex samples. The concept of using an array of cross-reactive sensors, a strategy mimicking the olfactory or gustatory systems of mammalians, takes this disadvantage as a benefit and, therefore, a new generation of sensing devices, which are capable of analyzing complex mixtures and have been developed [1]. 

E-nose technology, as a biomimetic approach, mimics the mammalian olfactory system [2]. It usually comprises two main components, the first being to provide an array of cross-reactive sensors (receptors) similar to olfactory receptors, and the next is the data-processing unit, which comprises different machine learning algorithms to predict, classify, and analyze various chemicals in vapor forms based on their chemical properties [3], similar to what happens in mammalians. Traditional E-noses based on analytical instruments have potential utilization in detecting trace amounts of volatile organic compounds (VOCs) but are restricted by their bulky size and high price, as well as a requirement for well-trained personnel to operate them [4]. Colorimetric sensor arrays (CSAs) or optical noses (O-noses), as new analogs of E-noses, have recently attracted a lot of interest within analytical chemistry for their interesting features, namely ease of fabrication, availability of a large amount of optically active reagents, and offering naked-eye inspection [5,6].

Optical nose devices are fabricated by depositing small volumes of reagent solution on a solid substrate, which is not only used to support the deposited reagents but plays a crucial role in the interaction of analytes with the receptors. Accordingly, choosing a suitable substrate is an important issue. Among the different employed substrates for making optical noses, for instance, paper, glass, and nylon, paper substrates have gained a lot of interest because of their great advantages, such as availability in different forms and shapes, low cost, ease of chemical surface modification, and the possibility of regulating its hydrophobic/hydrophilic properties. In this regard, paper-based sensors like the O-nose system have been developed as cheap, facile, disposable, and portable analytical tools [7,8]. In our research group, several studies have been investigated in paper-based O-noses in various applications [9,10,11,12,13,14,15].

Nowadays, nanomaterials, such as quantum dots (QDs), which exhibit unique electrical and optical properties, are emphasized [16]. There are many types of QDs, including silicon QDs [17], carbon QDs [18], graphene QDs [19], graphitic carbon nitride QDs [20], magnetic QDs [21], magneto fluorescent QDs [22], metal chalcogenides QDs [23], and so forth, which are drawing attention owing to their potential for sensing applications.

Among these, metal chalcogenide quantum dots (QDs) have a high value due to their unique and functional properties, related to both intrinsic (quantum confinement) and extrinsic (high-surface-area) effects, as dictated by their size, shape, and surface characteristics [23]. Thus, they have considerable promise for diverse applications, including energy conversion (thermoelectric and photovoltaics), photocatalysis, and sensing (electrochemical, optical, colorimetric, and so on) [24]. Also, the sensors comprising metal chalcogenide QDs mainly operate at room temperature. This is an advantage for the fabrication of simple and low-power-consumption gas sensors [25].

Naturally, the main part of an O-nose is its detection units. Researchers apply various strategies to enhance the O-nose’s performances (selectivity and sensitivity). Lin, Jang, and Suslick [26] innovated a considerable improvement in the sensitivity of colorimetric sensor arrays for the detection and identification of less reactive VOCs by using the pre-oxidation technique. The analyte’s stream was passed through a strong oxidant (e.g., chromic acid loaded on silica) before reaching the array. As a result, the analytes are converted into chemically reactive species, which have a stronger interaction with relevant colorimetric sensor elements. This technique and similar ones require complicated instrumentation and precise control of other parameters. 

A simpler strategy to enhance the sensitivity could be combining the sensitive receptors with nanostructures of some metal chalcogenide, such as molybdenum disulphide quantum dots (MoS_2_ QDs), due to their high surface area, high surface activity, and impressive optical and electronic properties [27,28,29]. MoS_2_ is known as an inorganic cousin of graphene, being rich in sulfur vacancies that provide them higher affinity to oxygen-functionalized VOCs [30]. In the past, MoS_2_ QDs have been used in E-nose systems for medical diagnosis [31] and food adulteration [13]. 

In our previous paper, by combining the catalytic properties of MoS_2_ QDs with the optical properties of acid based on indicators, we converted the E-nose feature MoS_2_ QDs to a paper-based O-nose system for the visual identification of some aldehydes and ketones. The fabricated opto-electronic nose was used for the analysis of formaldehyde as a toxic adulteration in milk samples [13]. 

In this work, we employed the above-mentioned O-nose device for the identification and discrimination of multiple cigarette smoke VOCs, such as alcohol, ester, and hydrocarbon (both aliphatic and aromatic), in addition to aldehydes and ketones. The secondary part of this study is to investigate whether the proposed O-nose is able to discriminate between original and fraudulent cigarette brands, as an environmental and health problem. Cigarette smoking can cause serious health risks, including, more importantly, lung cancer, insulin resistance, and abdominal-type obesity [32,33]. Cigarettes are composed of tobacco, with similar ingredients but different contents, which vary between brands and types of cigarettes. However, the chemical constituents of toxic chemicals contained in cigarette smoke and, consequently, the detrimental effects of smoking are affected by minute differences in cigarette ingredients [34,35]. 

On the other hand, fake duplicate cigarettes are a global issue [36]. Accordingly, the cigarette industry is losing millions of dollars per year due to fraudulent production [37]. Because of the complex nature of the chemical ingredients in tobacco, traditional analytical methods fail to investigate cigarette tobacco [38]. Alternatively, advanced instrumental methods, such as GC-MS and LC-MS, have been used for the analysis of cigarette tobacco. However, for the in-field and on-site analysis of cigarettes, simpler and portable devices such as E-nose systems are demanding. Using a biomimetic nose system based on colorimetric sensor arrays to analyze cigarettes is rarely reported, and nearly all reports are based on artificial E-nose sensing systems [36,39,40,41]. To the best of our knowledge, this work is the first report on using a paper-based O-nose for the analysis of cigarettes.

## 2. Experimental

### 2.1. Materials, Reagents, and Solutions

All the reagents used were of analytical grade without further purification. The chemicals used for synthesis of MoS_2_ QDs, including sodium molybdate dehydrate (Na_2_MoO_4_·2H_2_O), were obtained from Riedel-Dehaen AG, and L-cysteine was purchased from BDH. The optical sensing elements were pH indicators and fluorescent dyes, which were dissolved in MoS_2_ QD solution. Chlorophenol red (CPR), diamond fuchsin (DF), thymol blue (TB), diamine green (DG), phenol red (PR), and bromophenol blue (BPB) were provided by Merck. Methyl green (MG) and bromocresol green (BCG) were purchased from Sigma-Aldrich, and brilliant green (BG) was provided by Matheson Coleman and Bell company. The dye structures are shown in Table S1.

The cigarette smokes’ volatile organic compounds (VOCs) used in this study were acetophenone, acetaldehyde, crotonaldehyde, benzaldehyde, formaldehyde, anisaldehyde, ethylmethyl ketone, ethyl acetate, benzyl alchohol, toluene, ortho-xylene (o-xylene), 1-octene, ethyl benzene, and heptane, which were obtained from Merck Chemical Company. The VOCs are listed in Table S2.

### 2.2. Instruments and Software

Absorbance spectra were recorded using BEL-Gold-Spectrum-Lab 53 UV-Vis spectrophotometer equipped with a 1 cm quartz cell. Fluorescence measurements were conducted using a Perkin-Elmer LS50B fluorescence spectrophotometer with excitation and emission slits of 5 nm. An Elmasonic S 30 H ultrasonic was used for sonication of quantum dot solutions. HR-TEM images and energy-dispersive X-ray spectroscopy (EDS) analysis were recorded using FEI–TEC9G20 instrument (USA) at an accelerating voltage of 200 kV. Measurement of pH was performed using a Metrohm 632 pH meter (Model 780 pH lab). Spotting of reagent solution on the PVDF was carried out using a digital micropipette (BRAND Transferpette^®^ S, Wertheim, Germany). An oven (MEMMERT UN 30) was used for drying cigarette samples. Photographs of the sensor arrays were recorded using an ordinary flatbed scanner (CanoScanLiDE 700i, with a resolution of 600 dpi, New York, NY, USA). All calculations were conducted in Matlab R2018b (The MathWorks, Inc., Natick, MA, USA) in Microsoft Windows environment. The classification toolbox (version 5.3) developed by the Milano Chemometrics and QSAR Research Group was utilized for LDA classification, as well as for calculating model statistical parameters. For any updates regarding the toolbox, please refer to the following webpage: http://michem.disat.unimib.it/chm/ (accessed on 19 March 2022).

### 2.3. Synthesis of MoS_2_ QDs

In our previous paper [13], a hydrothermal method for the synthesis of water-soluble MoS_2_ QDs was described. Here, we provide a concise overview of the procedure. Initially, a 25.0 mL solution of 1.0% *w*/*v* Na_2_MoO_4_·2H_2_O in water was subjected to sonication for 5 min. The pH of the solution was adjusted to 6.5 by gradually adding 0.10 M HCl. Subsequently, a 50.0 mL solution of 1.0% *w*/*v* L-cysteine in water was introduced into the Na_2_MoO_4_ solution while gently stirring with a magnetic stirrer. The resulting mixture was then transferred to a 100 mL Teflon-lined stainless-steel autoclave and reacted at 200 °C for 36 h.

After cooling the solution in ambient temperature, the MoS_2_ QDs were separated by centrifuging at 12,000 rpm for 30 min. The supernatant containing the MoS_2_ QDs was collected, and it was subjected to dialysis using a dialysis membrane with a molecular weight cutoff (MWCO) of 3000 Da. The UV-visible absorbance, fluorescence spectroscopic methods, and HR-TEM analysis were employed for the characterization of the synthesized QDs.

### 2.4. Fabrication of MoS_2_ QD Sensor Array

A biomimetic sensing device with a 3 × 3 array format consisting of nine receptors was developed. The fabrication process involved mixing MoS_2_ QDs with acid-based organic reagents. Each organic reagent was added individually to a volumetric flask and diluted with the prepared solution of MoS_2_ QDs to achieve an indicator concentration of 0.01% *w*/*v*. At this concentration, the color stain of all nine organic reagents after drying on the PVDF substrate can be clearly seen with the naked eye.

**Scheme 1:**
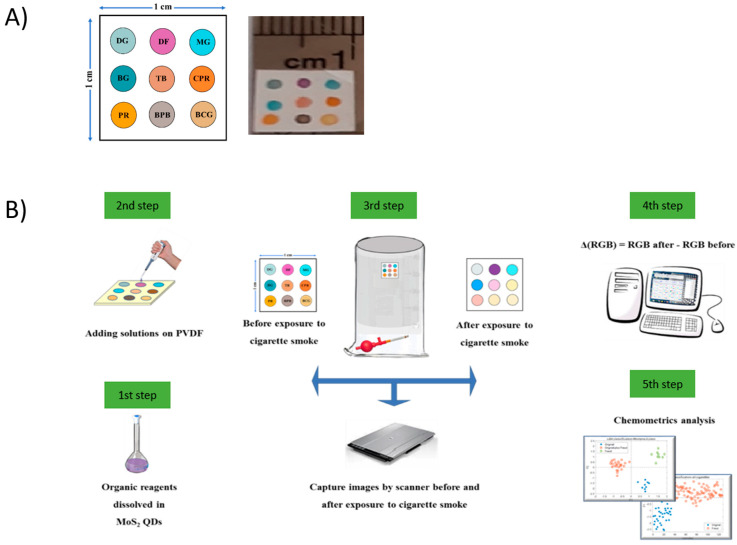
(**A**) Image of the fabricated O-nose device and (**B**) steps for fabricating O-nose device and measuring procedure.

However, if the concentration of organic reagents is high, the color changes caused by exposure to the analyte can hardly be observed. Through trial and error, and according to our previous experience with such sensing platforms [13], a concentration of 0.01 was suitable for all 9 reagents used in this study. The resultant mixtures were served as the receptor in the colorimetric sensor array. Spots were created on a PVDF film by depositing 0.20 µL portions of the QD–organic reagent mixture. The O-nose device was dried in a desiccator and stored in darkness before conducting sensing experiments. Detailed fabrication instructions are provided in Scheme 1.

### 2.5. Experimental Protocol for Study of Cigarette VOCs

To study VOCs of the cigarette smoke, a 500.0 µL portion of the analytes was poured into a petri dish without dilution or any other pretreatment. The fabricated MoS_2_ QD sensor was attached to the inside of petri dish’s cap. After the samples were transferred to the petri dish, its cap, holding the sensor array, was closed and sealed using parafilm. Measurements were conducted under the fuming hood for a specified time period (1 h) at room temperature. The space inside the petri dishes is limited. So, at ambient temperature, 1 h is enough that VOCs saturate the head space of the dish and establish the equilibria between the VOCs and the proposed sensor array. The image of MoS_2_ QD sensor array was recorded using a scanner before and after its exposure to the vapors of the samples. 

### 2.6. Cigarette Sample Preparation

A total of nine different brands of cigarette (five originals and four counterfeit) were obtained from local suppliers in Shiraz, Iran. The sampling program for collecting cigarettes used in the experiments covers several brands, differing by the type of tobacco leaves used in production, the technology, as well as the company of production. The original and counterfeit brands were provided under the supervision of the General Directorate of Tobacco in Fars Province (Shiraz, Iran). For each brand, three packets were purchased, and three cigarettes from the same batch were sampled from each packet. The paper and the vegetal portion of the cigarettes, i.e., tobacco, were analyzed, and filter was discarded. To partially equalize the humidity conditions of different cigarettes, prior to burning, they were kept in the oven at 40 °C for 15 min.

To investigate the effect of mixing substandard tobacco with high-quality tobacco content of the original brands, the tobacco content of both original and counterfeit cigarettes was removed from the cigarettes and ground separately in a mortar. Next, the two tobaccos with different quality were mixed together in various weight ratios. These mixtures were transferred into original brand paper and examined like other cigarettes. 

### 2.7. Conditions of Data Acquisition for Side-Stream Cigarette Smoke

The main objective of the experiment is to simplify the sampling methodology to collect side-stream cigarette smoke as much as possible without using sensor chamber and gas supply system with pumps and valves. One of the main reasons was reducing the cost and simulating the process of identifying the cigarette brands in situ without high-purity gas cylinders.

To burn a cigarette, the filter part of the cigarette was placed inside the sampling part of a rubber pipette filler. While the filler was in sampling mode, the other end of the cigarette was approached with a flame. Suction of air into the filler caused the cigarette to continue burning. Cigarette smoke was collected in the space above it, in a glass beaker (with 5 L volume), where the sensor was already stacked to its wall (Figure S1). The smoke was not diluted. The VOC products of burning the cigarettes and the sensor were in contact with each other at room temperature for 10 min. Then, the sensor was detached from the wall of the container and scanned to achieve the “after-exposure image”. The measuring procedure is depicted in Scheme 1.

### 2.8. Data Collection and Analysis

The images of the developed sensor array before and after exposure to the sample’s vapor were captured, and the differences in color intensities of the receptors between the before and after image (∆R, ∆G, and ∆B) were calculated and used to generate Color Difference Matrices (CDMs). Linear Discriminant Analysis (LDA) was used to classify and differentiate cigarette brands; also, identifying original and fraudulent products and the evaluation were performed through cross-validation.

## 3. Results and Discussion

### 3.1. Characterization of the Synthesized QDs 

Figure S2 presents HR-TEM images, showcasing the typical morphology of the synthesized MoS_2_ QDs. Energy-dispersive X-ray spectroscopy (EDS) analysis (Figure S3) confirmed the presence of Mo and S elements in the structure of the QDs. 

The optical properties of the MoS_2_ QDs were also investigated (Figure S4). Unlike some other quantum QDs, the MoS_2_ QDs exhibit a strong absorbance peak around 280 nm, with a slight shoulder in the 400–500 nm range. When irradiated at 300 nm, the MoS_2_ QD solution demonstrates intense blue fluorescence emission around 400 nm, as depicted in the inset images in Figure S4.

### 3.2. Humidity Tolerance and Durability of the Device

The development of an optical nose based on an MoS_2_ semiconductor addressed the issue of atmospheric humidity affecting electronic noses. In a previous study, the humidity effect was successfully diminished [13]. To further investigate the impact of humidity, the sensor array device was exposed to varying levels of relative humidity. The results showed that the developed MoS_2_ QD sensor array could tolerate up to 95% relative humidity without significant color changes in the sensing elements (Figures S5 and S6). These findings indicate that the sensor is independent of humidity, making it suitable for use in regions with fluctuating humidity levels.

In the case of practical applications, a long-term shelf life is also very important. To evaluate the stability of the developed MoS_2_ QD sensor array, the sensors were stored inside the desiccator after fabrication, without being exposed to analytes. It was scanned on different days, and its images were analyzed. The Euclidean distance diagram calculated for the sensor shows that the sensor has good stability for at least two weeks under normal storage conditions (Figure S7).

### 3.3. Device Response to Cigarette VOCs

**Figure 1:**
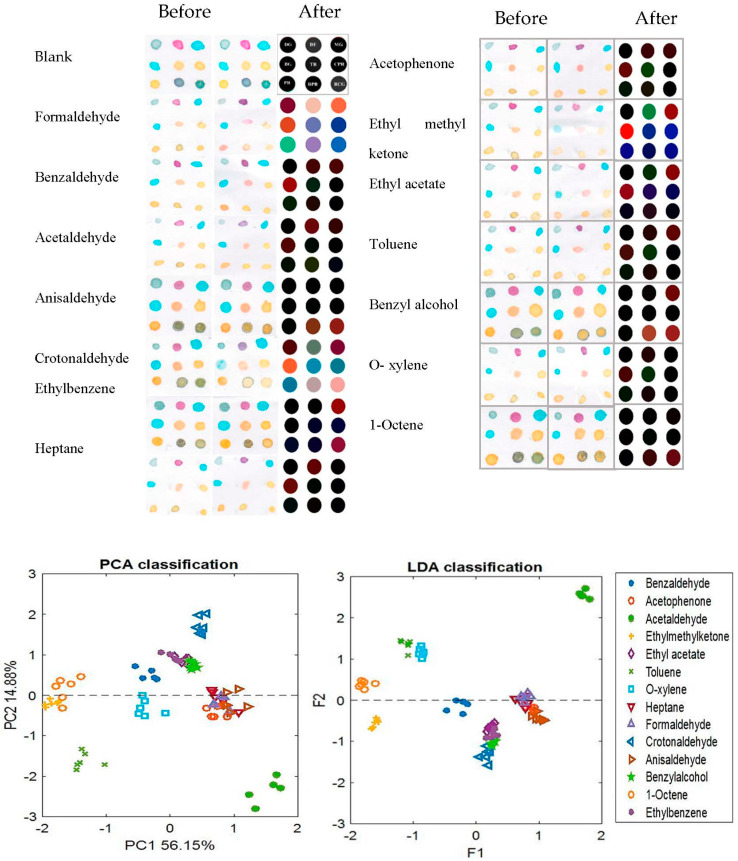
O-nose responses to different cigarette’s VOCs: before and after images of the O-nose response and their corresponding calculated CDMs; two-dimensional PCA score plot and LDA scatterplot for discriminating between 14 cigarette VOCs.

There are a large number of chemicals in cigarette smoke. Before studying the real cigarette samples, the potential of the O-nose for discriminating some cigarette VOCs was investigated. Herein, some chemicals with diverse functional groups, namely aliphatic and aromatic hydrocarbons, alcohols, esters, aldehydes, and ketones, with different carbon chain lengths, were studied (Section 2.5). Figure 1 shows the images of the sensor array before and after exposure to the vapor of the studied cigarette VOCs. The spots of the sensor array (colorimetric receptors) represented obvious but differential color changes. The collection of all receptors’ color changes was somehow unique for every studied analyte. The observed variations in the color intensities of the sensors of the same analyte can be attributed to between-sensor variations during fabrications of the sensor arrays, e.g., imprecise injection of reagents on the surface of PVDF paper. To decrease the effect of such variations, the images of sensors before exposure to the analyte’s vapors were also recorded, and color difference maps (CDMs) were generated, as explained in the Experimental section. As observed, CDMs show different patterns between different VOCs. It should be noted that CDMs were calculated based on the absolute values of the color differences, which means that, for two analytes of opposite effects but with the same intensities, the CDMs are the same. Therefore, for better understanding of the discrimination ability of the O-nose device, multivariate data analysis is required. 

Machine learning methods, which are inspired by the brain system of mammalians, are used to process the images of sensing devices and then to use these images for discriminating between different VOCs. Here, PCA and LDA, as supervised and unsupervised learning methods, were employed. In the lower-left panel of Figure 1, the distribution of the cigarette’s VOCs in a two-dimensional PCA score plot is shown. The PCA score plot shows fairly clear clustering of the data using only the first two principal components, explaining, cumulatively, 71% of the variances (PC1; 56.1% and PC2; 14.9%) in the original data matrix. When these PCs were fed into a discriminant model, accuracies of 96 and 86% were achieved for the training and cross-validation (venetian blinds approach), respectively. The corresponding statistical parameters are displayed in Table S3.

The LDA analysis was also performed on the raw data. The classification scheme is depicted in Figure 1 (lower-right panel), and the statistical parameters are presented in Table S4. In the 2D visual display, discrimination between VOCs cannot be evaluated, but statistical parameters show that the classification of VOCs was achieved. Compared to the PCA methods, LDA provided better results. The accuracy was 100% in both training and cross-validation sets. This demonstrates the high potential of the used cross-selective sensors for discriminating VOCs of different functional groups.

**Figure 2:**
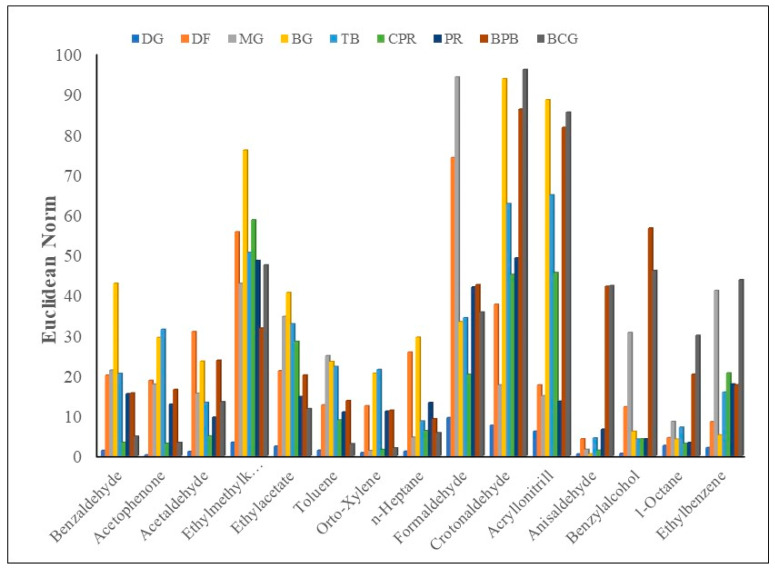
Bar plot showing the average response of the different sensing elements to the cigarette VOCs.

The cross-selectivity of the sensors used in this O-nose device is shown in Figure 2. The Euclidean norms of the RGB color intensity of every sensor are plotted. As observed, for none of the pairs of the studied analytes, the same signal intensities over all sensors were obtained. This numerical criterion was used to indicate the importance of each sensor in responding to the specific analyte. In the case of aliphatic and aromatic hydrocarbons, minimal color changes are created in the first sensor (DG). Almost all sensors significantly changed in response to toluene. In o-xylene, which differs from toluene only in one methyl group, the change in the third sensor (MG) shows a completely different pattern.

In the case of aliphatic aldehydes (formaldehyde and acetaldehyde), the color of the eighth (BPB) and ninth (BCG) sensors changed in the same manner. However, the third sensor (MG) could easily discriminate these aldehydes. The color changed to orange in the presence of formaldehyde, whereas acetaldehyde changed to dark brown in the CDMs. Among the other studied aldehydes, the fourth sensor (BG) has a different pattern. For anisaldehyde, the color of the fourth sensor did not change, while for crotonaldehyde, the color change was equivalent to orange on the CDM, and for benzaldehyde, the color change was equivalent to dark green on the CDM. Two completely different patterns were observed in ethyl methyl ketone (aliphatic) and acetophenone (aromatic) ketones. In total, all 14 VOCs were presented unique patterns, and this is proof of the ability of the MoS_2_ QD sensor array in discrimination between these cigarette VOCs.

### 3.4. Classification of the Cigarette Brands 

**Figure 3:**
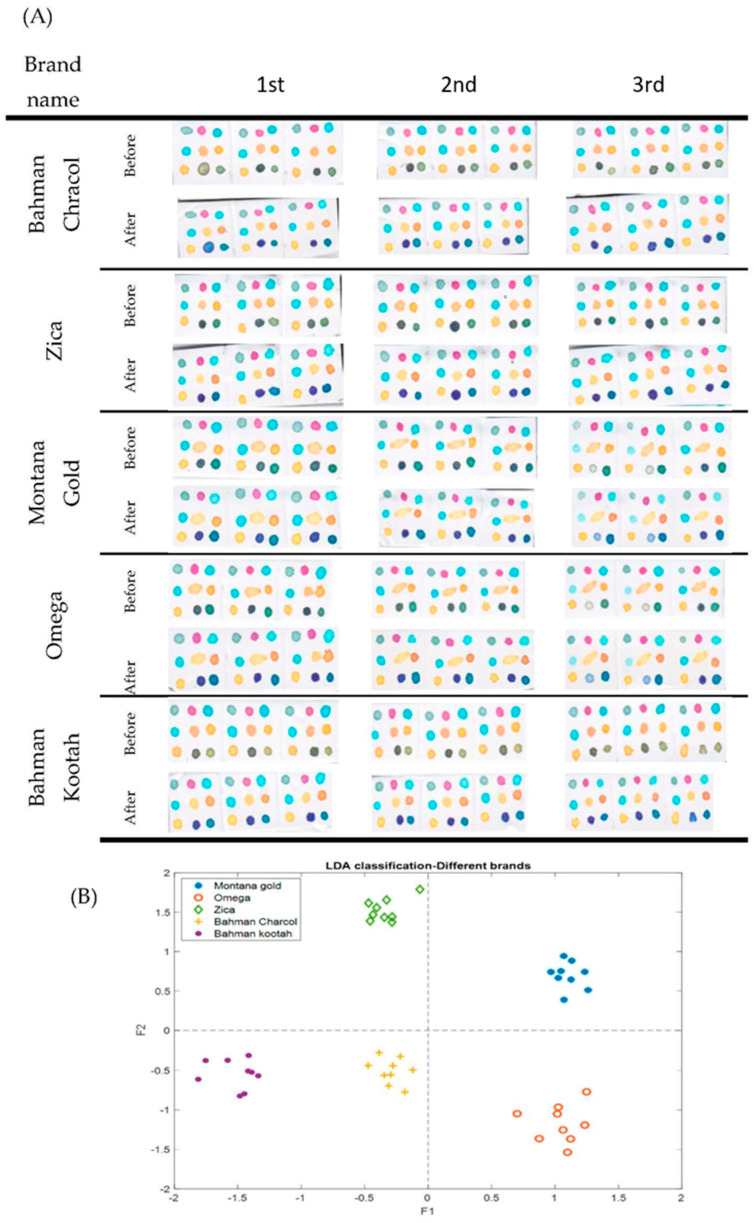
(**A**) Colorimetric response of the O-nose device to different cigarette brands. Every image of the samples composed of three parallel measurements. (**B**) The dissolution of the cigarette samples in the space of the LDA canonical variables.

In this study, the sensor was validated via the discrimination of five cigarette brands. By consulting the Directorate of Tobacco in Fars Province (Shiraz, Iran), the five top-used brands in Iran (three national brands, including Zica, Bahman charcoal, and Bahman Kootah, and two imported brands, namely Montana gold and Omega) were investigated. Each brand was sampled from three different markets at different time intervals, and each sample was measured three times. Therefore, in total, nine measurements were run for each brand and, totally, 45 samples were analyzed. The colorimetric responses of the arrays to all samples are shown in Figure 3. It is hard to conclude the discrimination of the cigarette brands via visual inspection. However, as shown in the lower panel of Figure 3, clear discrimination of the cigarette brands with 100% accuracy was achieved using LDA. It should be noted that the quality of the raw dataset was good enough that there was no need for any pre-processing steps. An interesting point is that the Iranian cigarettes were clearly discriminated from the imported ones in the direction of the first canonical variable.

### 3.5. Discrimination of the Original and Fraudulent Cigarette Brands

In the next step, four of the best-selling brands in the Iranian market (Bahman Kootah, Omega, Montana gold, and Williams), which are reported to have fraudulent samples (eight in total), were studied using the developed MoS_2_ QD sensor array. The appearance of the packaging and the shape and size of the cigarettes are quite similar and are indistinguishable to the average consumer. As mentioned earlier, these samples were prepared under the supervision of relevant government officials. The original and counterfeit samples were analyzed under exactly the same conditions. The calculated CDMs based on the sensors’ images are given in the Supplementary Materials section, Figure S8. Obviously, for the Bahman brand, the differences in the response of the O-nose device to the original and fraudulent samples are very significant; the fraud samples represented significant effects on the images of almost all sensing elements. However, for other brands, the differences between the fraud and original samples are not high for all sensing elements. Sensors 1 and 8 represented different color changes between the fraudulent and original samples for all brands. Other sensors exhibited slight differences in color changes.

**Figure 4:**
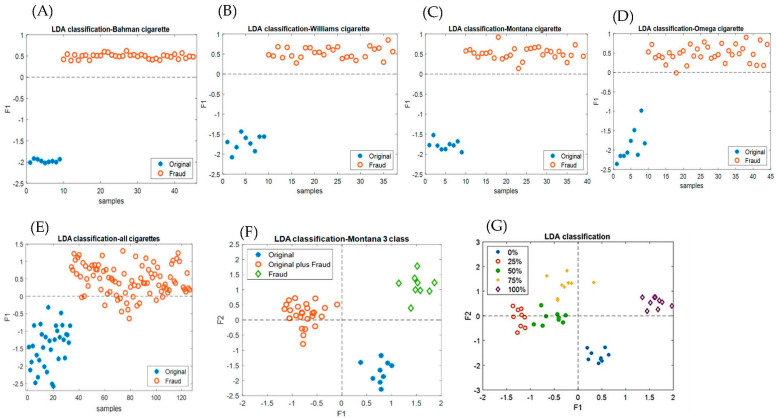
LDA canonical variate plots for discrimination of the fraud and original cigarettes of four brands individually (**A**) Bahman, (**B**) Williams, (**C**) Montana, and (**D**) Omega. (**E**) Discrimination of the fraud and original cigarettes of all brands, (**F**) LDA analysis for identification of low-quality fraud tobacco, and (**G**) LDA scatter plot for identification of low-quality fraud tobacco in different percentage.

The raw color difference data were examined using the LDA learning method. Firstly, four brands, which have fraudulent samples, including Bahman (Figure 4A), Williams (Figure 4B), Montana (Figure 4C), and Omega (Figure 4D), were investigated separately; for every brand, a separate discriminant model was built. As shown in Figure 4A–D, the fraud samples were significantly differentiated from the original samples; all brands had an accuracy of 100%. 

In another study, all original and fraudulent samples (126 cigarettes) were analyzed in a matrix with dimensions of (126 × 27) using LDA. Figure 4E shows the discrimination between original and fraudulent samples. The accuracy of this LDA model was 98%. As it turns out, the developed MoS_2_ QD sensor array was well able to recognize fraudulent cigarettes (statistical parameters are listed in Table S5).

Mixing high-quality tobacco with additives, such as paper, wood chips, or low-quality tobacco, is another type of fraudulent cigarette. To show the potential of the O-nose device to identify this kind of adulteration, the Montana brand, for which a fraudulent counterpart is available on the market, was chosen. The contents of the cigarettes were emptied. The original sample tobaccos were then mixed with different weight percentages of the fraudulent cigarette content. Eventually, these mixtures were refilled into the original cigarette paper with the same initial weight. The response of the O-nose device to original, fraudulent, and original–fraud mixtures was recorded and analyzed using LDA. The distribution of the objects in the two-dimensional LDA scores is given in Figure 4F. Obviously, the O-nose device was able to accurately identify the fraudulent cigarettes (both pure fraud and their mixtures with original) from the original one. The accuracy of the LDA model was 100% for the training set and 90% for the cross-validation set (Table S6).

Finally, the potential of the sensor for determining the amount of the added counterfeit samples was investigated. The mixture samples contained 25%, 50%, and 75% added fraudulent cigarettes to the original one. Therefore, in total, 5% compositions (0, 25, 50, 75, and 100) were available, and a 5-class LDA model was run. As given in Figure 4G, the sensor was able to discriminate the mixed samples of different percent compositions. However, the complete discrimination is not evident from the shown plot, and the mixed classes represented some overlaps.

## 4. Conclusions

This study reports the application of a newly designed colorimetric sensor array based on MoS_2_ QDs to investigate the authenticity of cigarettes. The optical sensing device, which worked based on the olfactory system of mammalians, sniffs the cigarette smoke and then classifies it based on the brand or the quality of the filled tobacco. The scope of its application was considerably expanded from simple compounds (organic solvents) to very complex chemical matrices, presented, herein, as cigarette side-stream smoke. There was no need for a pre-processing step in the data analysis due to the good quality of the data obtained using the developed MoS_2_ QD sensor array. A special side-stream smoke analysis system was also built for distinguishing between cigarettes of different brands with high accuracy. 

The analytical procedure proposed here is fast, cheap, user-friendly, and reliable. The developed MoS_2_ QD sensor array used portable devices, allowing for in situ quality control. The selectivity achieved broadens the application of this system to a great variety of brands and could make this method a valuable tool to differentiate original cigarettes from counterfeit products crossing national borders around the world every day.

## Supplementary Materials

The following supporting information can be downloaded at: https://www.mdpi.com/article/10.3390/bios13070705/s1, Table S1. The chemical formula and structure of the dyes employed in the fabrication sensor ar-ray. Table S2. The structure and classification of investigated of cigarette smoke volatile compounds. Table S3. Statistic parameters for PCA analysis for 14 VOCs via utilization of the response intensity of the MoS_2_ QDs sensor array. Table S4. Statistic parameters for LDA analysis for 14 VOCs via utilization of the response intensity of the MoS_2_ QDs sensor array. Table S5. Statistical parameters of LDA analysis of different original brands via utilization of the response intensity of the MoS_2_ QDs sensor array. Table S6. Statistic parameters related to LDA scatter plot of discrimination of different original brands via utilization of the response intensity of the MoS_2_ QDs sensor array. Table S7. Statistic parameters of LDA analysis of original and fraud samples. Table S8. Statistical parameters of LDA study of low quality fraud tobacco in cigarettes via utili-zation of the response intensity of the MoS_2_ QDs sensor array. Table S9. Experimental condition for study tobacco quality. Table S10. Statistical parameters for LDA analysis of tobacco fraud percentage via utilization of the response intensity of the MoS_2_ QDs sensor array. Figure S1. Cigarette smoke sampling. Placing the sensor array inside the wall of the container (left), and sensor exposure to burning cigarette smoke (right). Figure S2. HR-TEM images of the synthesized MoS_2_ QDs with two different magnifications. Dis-play of the present of polyhedral particles (a), and white particles on a hexagonal particle’s sur-face (b). Figure S3. Energy-dispersive X-ray spectroscopy (EDS) of the synthesized MoS_2_ QDs. Figure S4. UV-Vis and fluorescence spectra of MoS_2_ QDs. The intercept is image of MoS_2_ QDs so-lution under UV irradiation (280 nm). Figure S5. Sensor response in different humidity between 10 to 95%. Figure S6. Plot of the RGB changes in different humidity (10–95%). Figure S7. Durability of fabricated MoS_2_ QDs sensor array. Figure S8. CDMs for four original and for fraud brands via utilization of the response intensity of the MoS_2_ QDs sensor array.
