# Supplementary material for: A Paper-Based Biomimetic Sensing Device for the Discrimination of Original and Fraudulent Cigarette Brands Using Mixtures of MoS2 Quantum Dots and Organic Dyes
Source: Biosensors (Basel). 2023 Jul 4;13(7):705. doi: 10.3390/bios13070705 (PMC10377080; doi:10.3390/bios13070705)
Supplement: Supplementary file 1 [file biosensors-13-00705-s001.zip › biosensors-2426871-supplementary.pdf]

biosensors

Article

A Paper-Based Biomimetic Sensing Device for the Discrimination of Original and Fraudulent Cigarette Brands Using Mixtures of MoS_2 Quantum Dots and Organic Dyes

Fereshte Mohamadi Gharaghani [1], Sara Mostafapour [1] and Bahram Hemmateenejad [1,2,*]

1 Chemistry Department, Shiraz University, Shiraz 71456, Iran; fereshtemohamadi.chem@gmail.com (F.M.G.); mostafapoursara69@gmail.com (S.M.)
2 Medicinal and Natural Products Chemistry Research Centre, Shiraz University of Medical Sciences, Shiraz 7134853734, Iran
* Correspondence: hemmatb@shirazu.ac.ir

Table S1. The chemical formula and structure of the dyes employed in the fabrication sensor array.

Compound Name	Chemical Formula	Chemical Structure
Chlorophenol red (CPR)	$C_{19}H_{12}Cl_2O_5S$	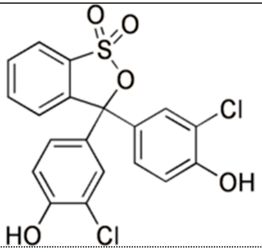
Bromocresol green (BCG)	$C_{21}H_{14}Br_4O_5S$	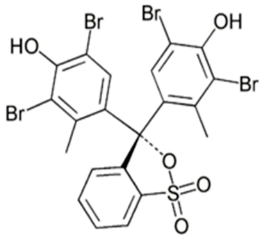
Diamond fuchsin (DF)	$C_{20}H_{19}N_3{\cdot}HCl$	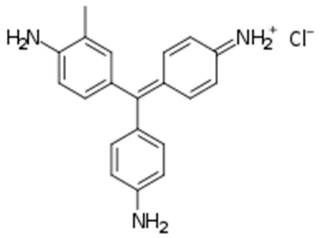
Thymol blue (TB)	$C_{27}H_{30}O_5S$	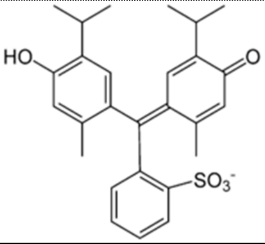
Diamine green (DG)	$C_{34}H_{22}N_8Na_2O_{10}S_2$	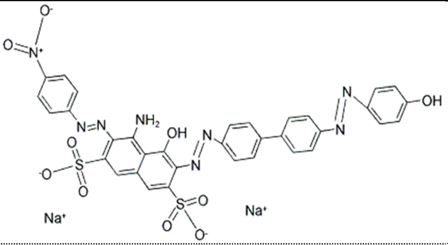
Bromophenol blue (BPB)	$C_{19}H_{10}Br_4O_5S$	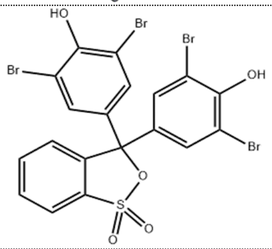
Phenol red (PR)	$C_{19}H_{14}O_5S$	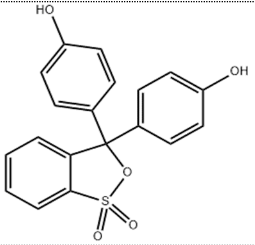

Methyl green (MG)	$C_{26}H_{33}Cl_2N_3$	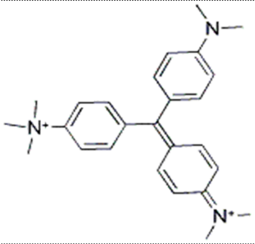
Brilliant green (BG)	$C_{27}H_{33}N_2.HO_4S$	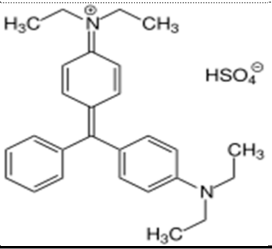

Table S2. The structure and classification of investigated of cigarette smoke volatile compounds.

Family	Compound name	Structure
**Alcohol**	Benzyl alcohol	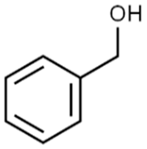
**Ester**	Ethyl acetate	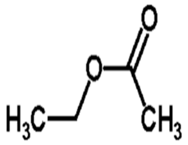
**Aromatic hydrocarbon**	Ethylbenzene	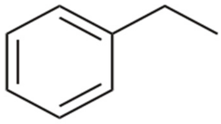
	Toluene	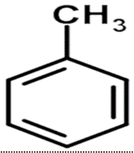
	Ortho- xylene	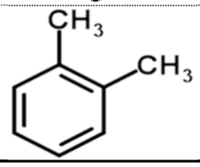
**Ketone**	Ethyl methyl ketone	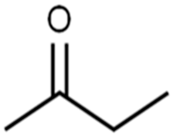
	Acetophenone	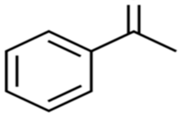

**Aldehyde**	Formaldehyde	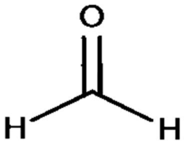
	Acetaldehyde	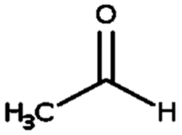
	Crotonaldehyde	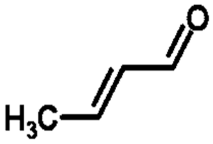
	Benzaldehyde	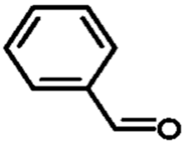
	Anisaldehyde	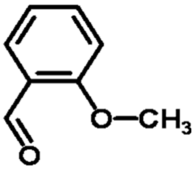
**Hydrocarbon**	1-Octene	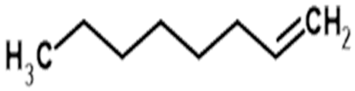
	Heptane	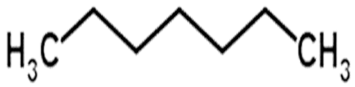

Table S3. Statistic parameters for PCA analysis for 14 VOCs via utilization of the response intensity of the MoS_2 QDs sensor array.

Parameters	Sensitivity		Specificity		Precision	
	Training	CV	Training	CV	Training	CV
Class1	1.00	1.00	1.00	1.00	1.00	1.00
Class2	1.00	0.83	0.99	0.99	0.86	0.83
Class3	1.00	1.00	1.00	1.00	1.00	1.00
Class4	1.00	0.83	1.00	0.97	1.00	0.71
Class5	1.00	0.83	1.00	0.99	1.00	0.83
Class6	1.00	1.00	1.00	1.00	1.00	1.00
Class7	1.00	1.00	1.00	1.00	1.00	1.00
Class8	0.83	0.67	1.00	0.99	1.00	0.80
Class9	0.80	0.60	0.99	0.97	0.80	0.60
Class10	1.00	1.00	1.00	1.00	1.00	1.00
Class11	0.83	0.83	0.99	0.97	0.83	0.71
Class12	1.00	0.83	1.00	0.99	1.00	0.83
Class13	1.00	0.67	1.00	0.99	1.00	0.80
Class14	1.00	1.00	1.00	1.00	1.00	1.00

*CV: Cross-validation.

Table S4. Statistic parameters for LDA analysis for 14 VOCs via utilization of the response intensity of the MoS_2 QDs sensor array.

Parameters	Sensitivity		Specificity		Precision	
	Training	CV	Training	CV	Training	CV
Class1	1.00	1.00	1.00	1.00	1.00	1.00
Class2	1.00	1.00	1.00	1.00	1.00	1.00
Class3	1.00	1.00	1.00	1.00	1.00	1.00
Class4	1.00	1.00	1.00	1.00	1.00	1.00
Class5	1.00	1.00	1.00	1.00	1.00	1.00
Class6	1.00	1.00	1.00	1.00	1.00	1.00
Class7	1.00	1.00	1.00	1.00	1.00	1.00
Class8	1.00	1.00	1.00	1.00	1.00	1.00
Class9	1.00	1.00	1.00	1.00	1.00	1.00
Class10	1.00	1.00	1.00	1.00	1.00	1.00
Class11	1.00	1.00	1.00	1.00	1.00	1.00
Class12	1.00	1.00	1.00	1.00	1.00	1.00
Class13	1.00	1.00	1.00	1.00	1.00	1.00
Class14	1.00	1.00	1.00	1.00	1.00	1.00

*CV: Cross-validation.

Table S5. Statistical parameters of LDA analysis of different original brands via utilization of the response intensity of the MoS_2 QDs sensor array.

Parameters	Sensitivity		Specificity		Precision	
	Training	CV	Training	CV	Training	CV
Class1	1.00	0.89	1.00	1.00	1.00	1.00
Class2	1.00	1.00	1.00	1.00	1.00	1.00
Class3	1.00	0.78	1.00	0.92	1.00	0.70
Class4	1.00	0.67	1.00	0.94	1.00	0.75
Class5	1.00	0.78	1.00	0.92	1.00	0.70

*CV: Cross-validation.

Table S6. Statistic parameters related to LDA scatter plot of discrimination of different original brands via utilization of the response intensity of the MoS2 QDs sensor array.

Plot	Parameters	Sensitivity		Specificity		Precision	
		Training	CV	Training	CV	Training	CV
A	Class 1	1.00	1.00	1.00	1.00	1.00	1.00
	Class 2	1.00	1.00	1.00	1.00	1.00	1.00
B	Class 1	1.00	0.89	1.00	0.94	1.00	0.80
	Class 2	1.00	0.94	1.00	0.89	1.00	0.97
C	Class 1	1.00	1.00	1.00	0.86	1.00	0.64
	Class 2	1.00	.86	1.00	1.00	1.00	1.00
D	Class 1	1.00	1.00	1.00	0.82	1.00	0.64
	Class 2	1.00	0.82	1.00	1.00	1.00	1.00

*CV: Cross-validation.

Table S7. Statistic parameters of LDA analysis of original and fraud samples.

Parameters	Sensitivity		Specificity		Precision	
	Training	CV	Training	CV	Training	CV
Class1	0.94	0.85	0.99	0.97	0.97	0.90
Class 2	0.99	0.97	0.94	0.85	0.98	0.95

*CV: Cross-validation.

Table S8. Statistical parameters of LDA study of low quality fraud tobacco in cigarettes via utilization of the response intensity of the MoS_2 QDs sensor array.

Parameters	Sensitivity		Specificity		Precision	
	Training	CV	Training	CV	Training	CV
Class1	1.00	1.00	1.00	0.89	1.00	0.69
Class2	1.00	0.85	1.00	1.00	1.00	1.00
Class3	1.00	0.89	1.00	0.97	1.00	0.89

*CV: Cross-validation.

Table S9. Experimental condition for study tobacco quality.

Experimental Set	Number of Repeat	Original Tobacco (% W/W)	Fraud Tobacco (% W/W)
1	9	100	0
2	9	75	25
3	9	50	50
4	9	25	75
5	9	0	100

Table S10. Statistical parameters for LDA analysis of tobacco fraud percentage via utilization of the response intensity of the MoS_2 QDs sensor array.

Parameters	Sensitivity		Specificity		Precision	
	Training	CV	Training	CV	Training	CV
Class1	1.00	1.00	1.00	0.94	1.00	0.82
Class2	1.00	0.67	1.00	0.86	1.00	0.55
Class3	1.00	0.56	1.00	0.92	1.00	0.63
Class4	1.00	0.33	1.00	0.86	1.00	0.38
Class5	1.00	0.67	1.00	0.97	1.00	0.86

*CV: Cross-validation.

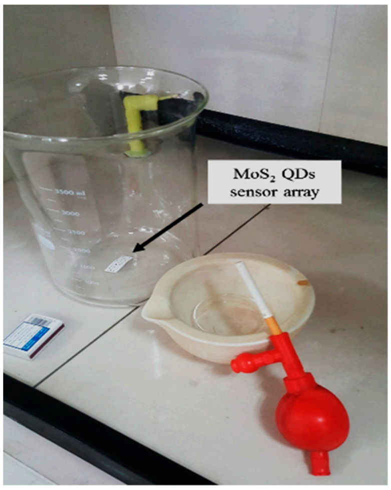

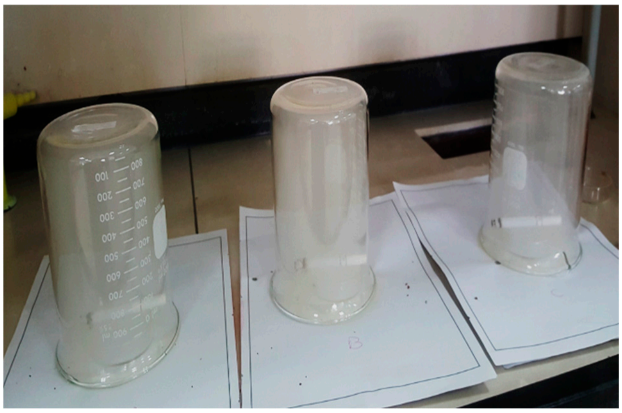

Figure S1. Cigarette smoke sampling. Placing the sensor array inside the wall of the container (**left**), and sensor exposure to burning cigarette smoke (**right**).

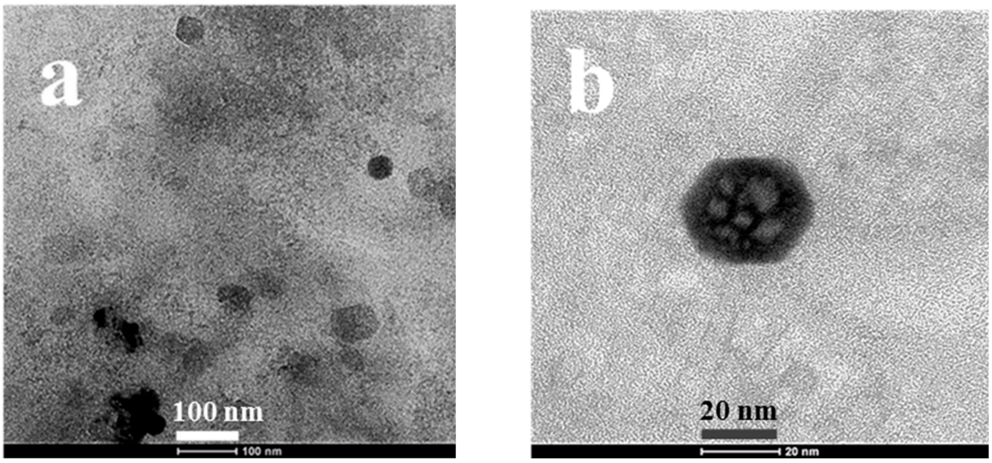

Figure S2. HR-TEM images of the synthesized MoS_2 QDs with two different magnifications. Display of the present of polyhedral particles (**a**), and white particles on a hexagonal particle's surface (**b**).

Figure S2 shows the typical HR-TEM images of the synthesized MoS_2 QDs with two different magnifications. Figure S2a shows a set of polyhedral particles, probably the NaCl crystals that accompany QDs as a by-product in the synthesis procedure and white particles on its surface are assumed MoS_2 QDs. Figure S2b shows that the size of these white particles is under 10 nm.

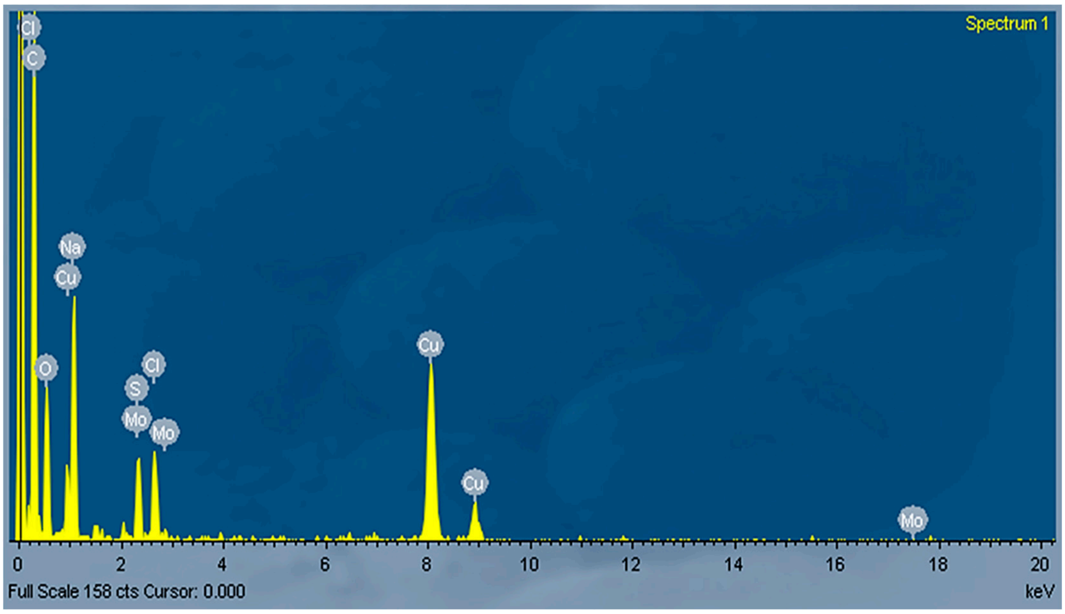

Figure S3. Energy-dispersive X-ray spectroscopy (EDS) of the synthesized MoS_2 QDs.

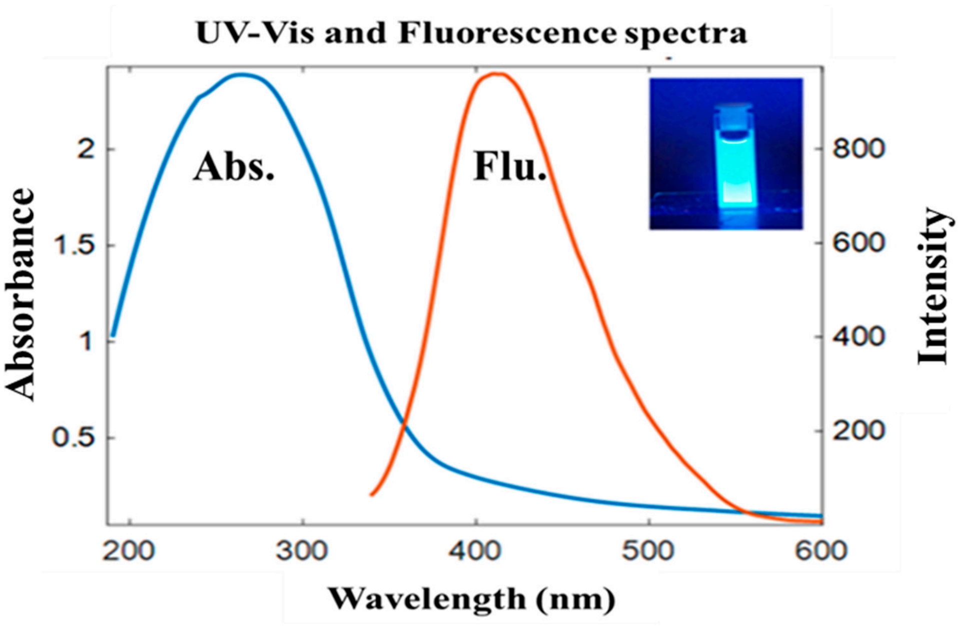

Figure S4. UV-Vis and fluorescence spectra of MoS_2 QDs. The intercept is image of MoS_2 QDs solution under UV irradiation (280 nm).

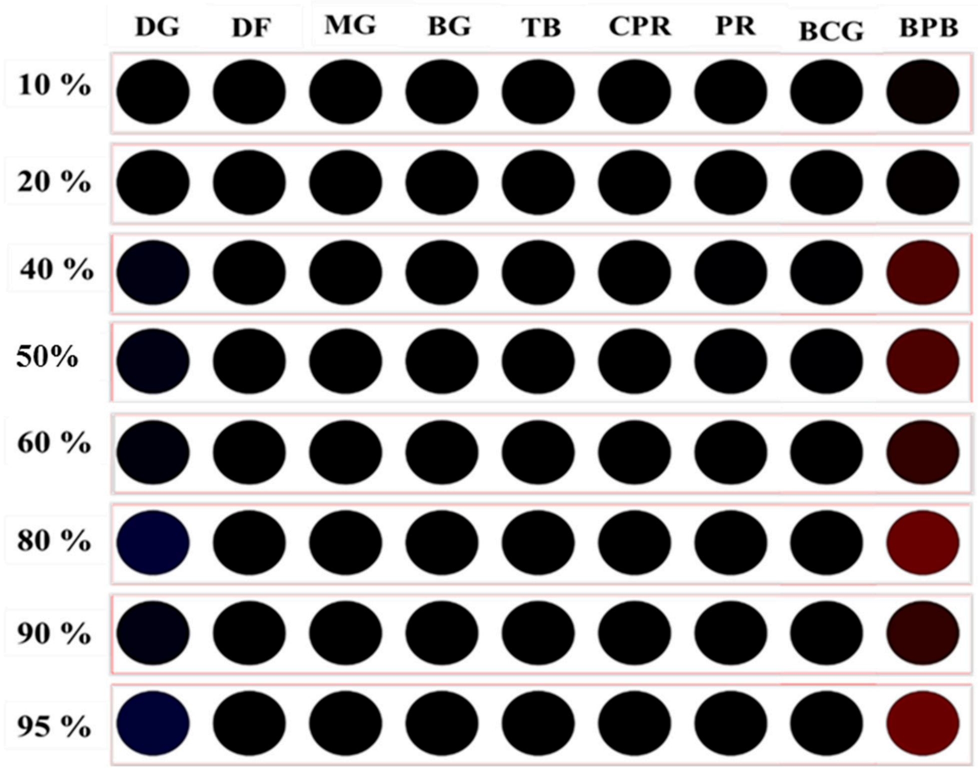

Figure S5. Sensor response in different humidity between 10 to 95%.

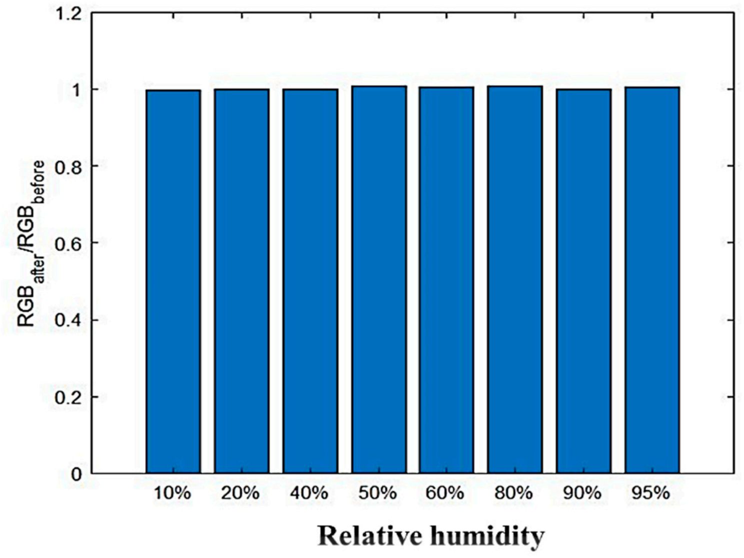

Figure S6. Plot of the RGB changes in different humidity (10-95 %).

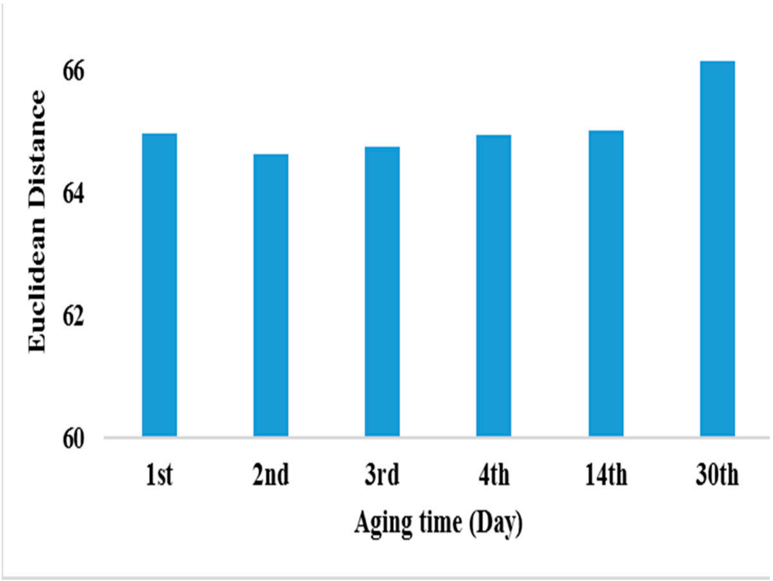

Figure S7. Durability of fabricated MoS_2 QDs sensor array.

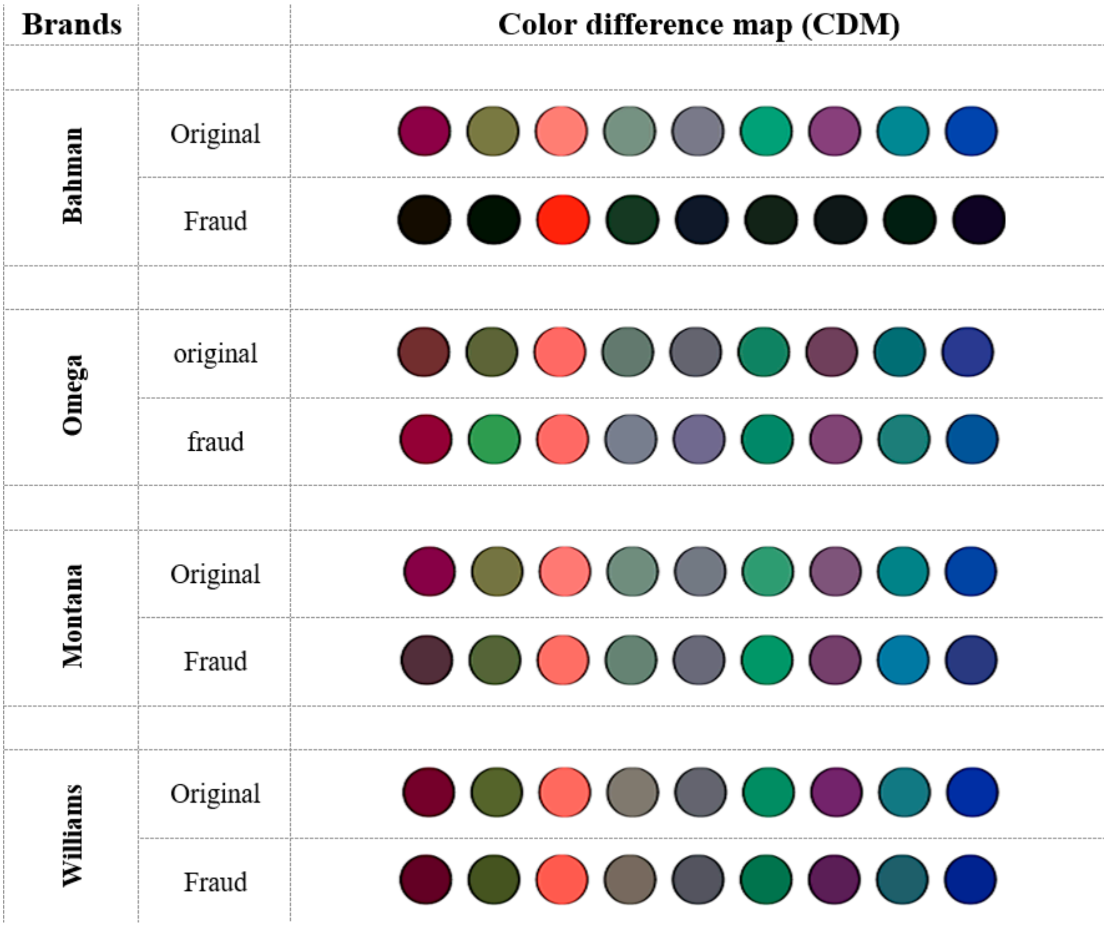

Figure S8. CDMs for four original and for fraud brands via utilization of the response intensity of the MoS_2 QDs sensor array.
